# Primary cardiac tumor: a case report of right atrial angiosarcoma and review of the literature

**DOI:** 10.3389/fonc.2023.1164153

**Published:** 2023-05-26

**Authors:** Yujian Guo, Qianzhen Liu, Haibo Wu

**Affiliations:** ^1^ Department of Changzhi People's Hospital, The Affiliated Hospital of Changzhi Medical College, Changzhi, Shanxi, China; ^2^ Department of Pathology, The First People’s Hospital of Foshan, Foshan, Guangdong, China; ^3^ Department of Cardiothoracic Surgery, Changzhi People’s Hospital, Changzhi, Shanxi, China

**Keywords:** primary cardiac tumors, primary cardiac angiosarcoma, angiosarcoma, right atrium, pericardial effusion

## Abstract

Primary cardiac angiosarcoma is a relatively rare tumor with early metastasis and poor prognosis. Radical resection of the primary tumor remains the primary approach for the optimal survival of patients with early-stage cardiac angiosarcoma without evidence of metastasis. This case involves a 76-year-old man with symptoms of chest tightness, fatigue, pericardial effusion, and arrhythmias who achieved good results after surgery to treat the angiosarcoma in the right atrium. In addition, literature analysis showed that surgery remains an effective way of treating primary early angiosarcoma.

## Introduction

Primary cardiac tumors are exceedingly rare and detected in 0.002% to 0.33% of autopsies (1, 2), with 0.15% revealed by echocardiography (3). Benign tumors are more common than malignant growths (4). Approximately 20% to 30% of all primary cardiac tumors are malignant, including angiosarcoma, lymphoma, fibrosarcoma, myosarcoma, and myxosarcoma (1). Of these, angiosarcoma is the most common type of primary cardiac malignancy in adults, accounting for 28.6% of these malignant cardiac tumors (4, 5). Cardiac angiosarcoma originates from endothelial cells and occurs mostly in men aged 30–50 (3, 6, 7). The survival rate of patients with cardiac angiosarcoma is generally reduced due to the unique location and aggression of the tumor, as well as late detection, challenging surgical procedures, and incomplete surgical resection (8). Radical resection of the primary tumor remains an effective strategy for achieving optimal survival (7).

## Case report

**Figure 1:**
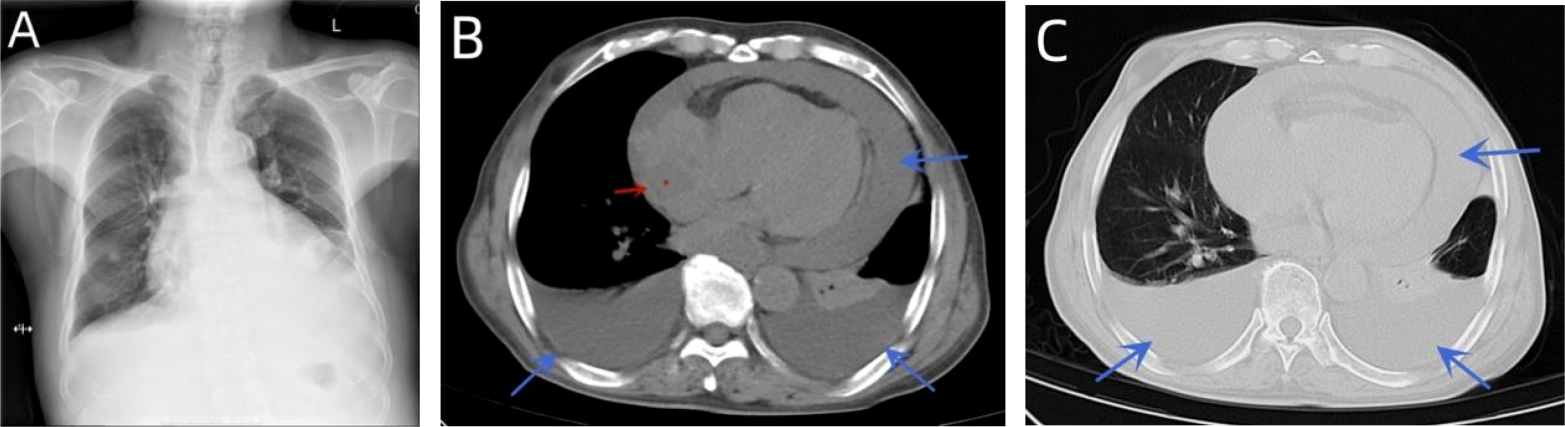
A chest X-ray showed an enlarged heart shadow and possible left pleural enveloping effusion **(A)**. CT indicated curved fluid density shadows in the bilateral thorax and pericardial cavity and irregular, slightly low-density shadows in the right atrium **(B, C)**.

**Figure 2:**
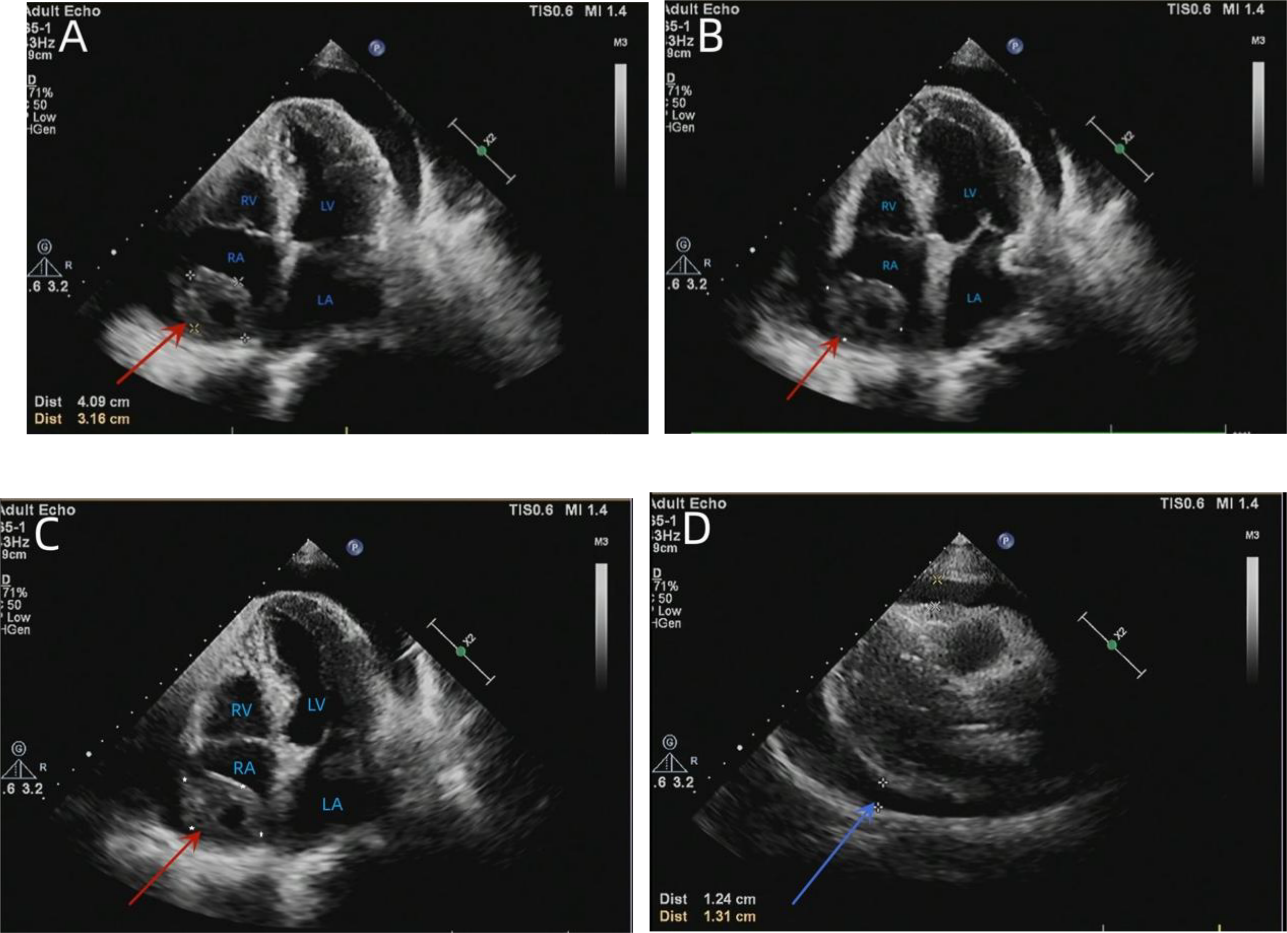
TTE showed an isoechoic mass of approximately 41 * 32 mm in the right atrium, with a broad base, multiple echo-free zones, and thickened surrounding chamber walls **(A–C)**. A dark area of fluid was visible in the pericardial cavity, and the fluid area was clear **(D)**. TTE, transthoracic echocardiography.

A 76-year-old male patient had been suffering from chest tightness, fatigue, cough, and sputum (white mucus sputum) for 3 weeks, with no obvious fever. Before presentation to our hospital, the patient visited a hospital nearby. Upon examination, a chest X-ray showed an enlarged heart shadow and possible left pleural enveloping effusion (**Figure 1A**). According to the computed tomography (CT) report, an irregular, slightly low-density shadow in the right atrium displayed middle-volume bilateral pleural and pericardial effusion (**Figures 1B, C**). Transthoracic echocardiography (TTE) showed a mass in the right atrium (approximately 41 * 32 mm) and fluid effusion in the pericardium (moderate volume) (**Figures 2A-D**). In addition, the plasma D-dimer [D-Dimer] concentration was 2,816.00 ng/ml (normal < 243). The patient was taken to the hospital for further treatment. He had a history of hypertension for more than 10 years and regularly took sustained-release nifedipine tablets. Physical examination revealed a malnourished man in acute distress with a regular pulse rate of 130 beats/min and weak heart sounds. His laboratory test results included a whole blood C-reactive protein [CRP] concentration of 24.16 mg/L, a serum troponin I [hsTnI] concentration of 11.90 ng/L, and an N-terminal-B-type natriuretic peptide precursor [NT-proBNP] level of 245.9 pg/ml. Electrocardiography (ECG) showed frequent premature atrial contractions and a rapid ventricular rate atrial flutter.

**Figure 3:**
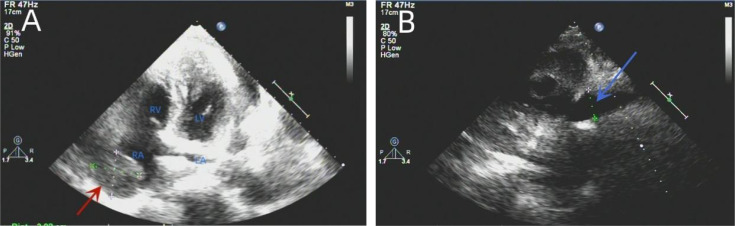
Re-examination of the TTE showed an isoechoic mass of approximately 39 * 37 mm at the top of the right atrium with a wide base and multiple echo-free zones. The surrounding atrial wall was thickened, and the width of the inferior vena cava was approximately 23 mm **(A)**. A dark area of fluid was visible in the pericardial cavity, and the fluid area was not clear **(B)**. TTE, transthoracic echocardiography.

**Figure 4:**
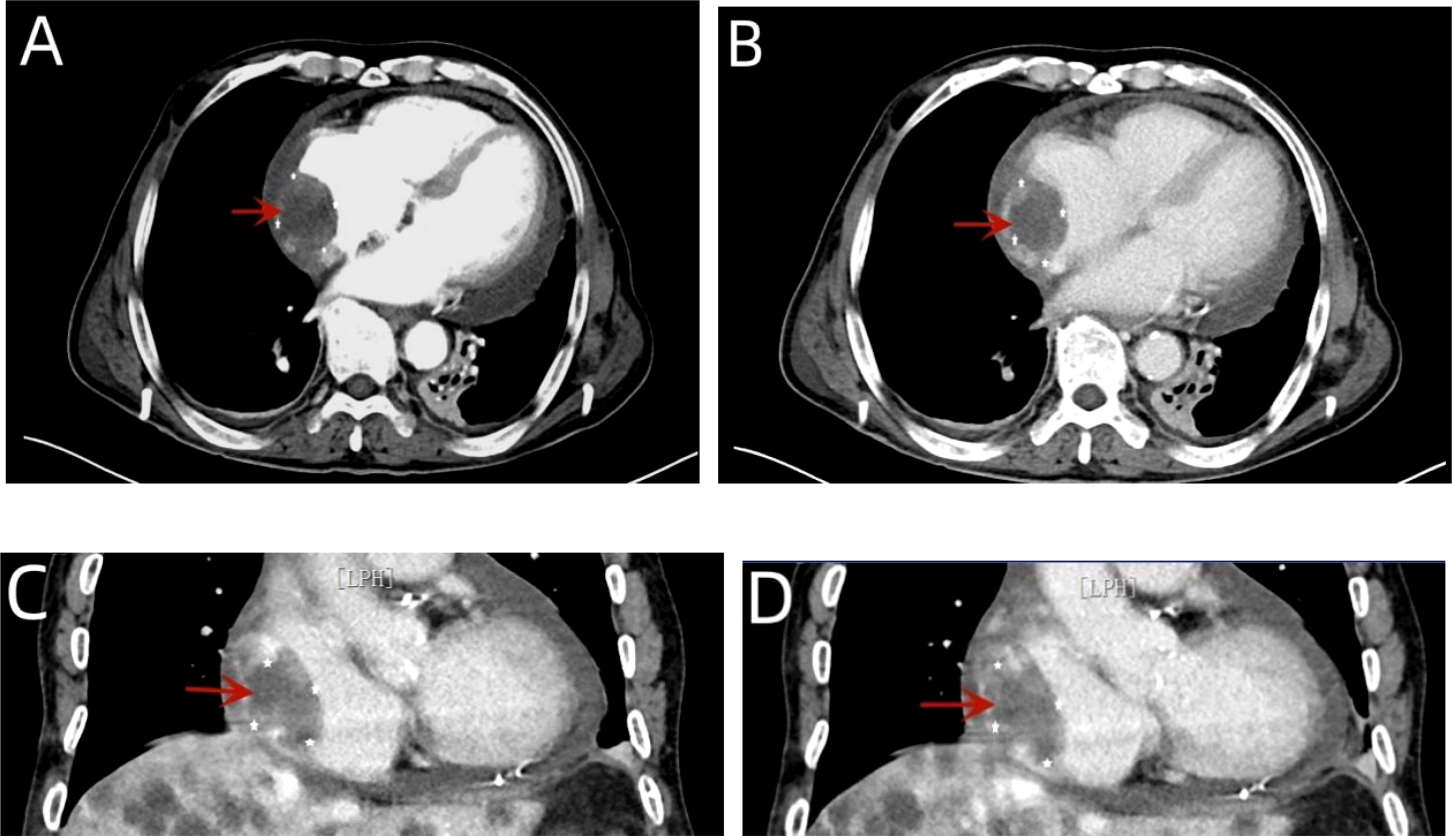
A contrast-enhanced CT of the chest showed arc-shaped fluid density shadows in the bilateral thorax and pericardial cavity, while irregular, slightly low-density shadows with unclear boundaries were evident in the right atrium (red arrow). Most of the lesions were not enhanced, and the edges appear locally enhanced **(A–D)**.

Next, thoracocentesis was performed on the patient, revealing a yellowish effusion discharging from the drainage tube. The patient’s chest tightness improved after pericardiocentesis. Although the pleural and pericardial effusions contained lymphocytes, histiocytes, mesothelial cells, and neutrophils, no clear malignant cells were evident. TTE re-examination showed an isoechoic, wide-based mass of approximately 39 * 37 mm at the top of the right atrium, which was considered the right atrial mass (**Figure 3A**). In addition, moderate-massive effusion was evident in the pericardium (**Figure 3B**). A contrast-enhanced CT of the chest and abdomen indicated an irregular, slightly low-density shadow with unclear boundaries in the right atrium (**Figures 4A-D**). Considering the patient’s critical condition and poor physique, surgery was eliminated as an immediate option, instead providing symptomatic treatment, such as fluid rehydration, oxidative atomization, and anti-infection. Intensive nutritional support relieved the patient’s symptoms, and he was discharged.

**Figure 5:**
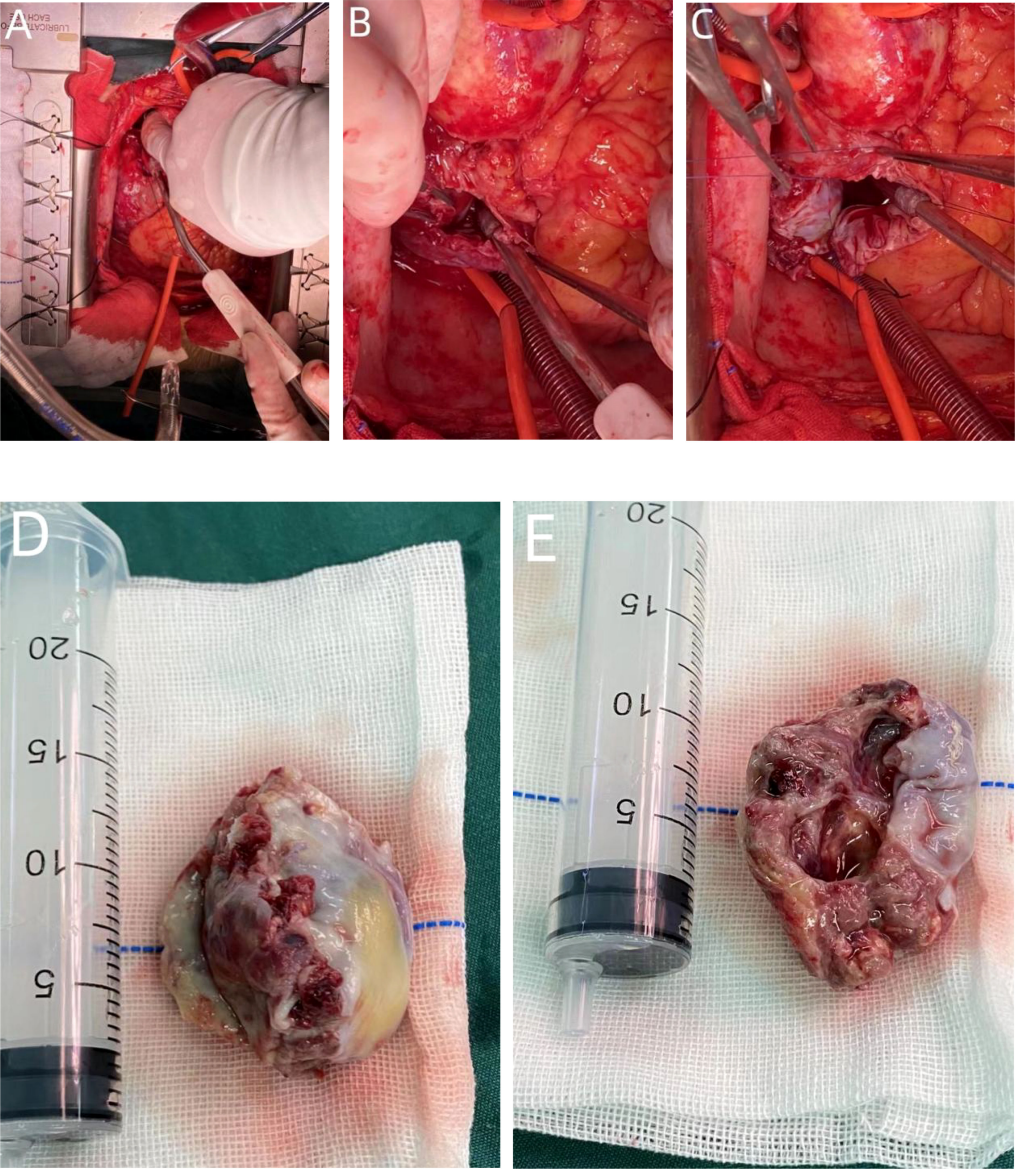
During the operation, a tumor protrusion of approximately 10 * 15 mm was evident on the surface of the right atrium. The serosal surface of the right atrium and superior vena cava showed cowhide changes **(A)**. Although the mass proliferated between the inner and outer membranes of the right atrial wall, it did not breach the atrial cavity **(B)**. The size of the mass was approximately 50 * 40 mm. The tumor morphology was considered for atrial sarcoma diagnosis **(D, E)**. The tumor was completely removed, as well as part of the atrial wall, and the right atrial defect was sutured with a pericardial patch **(C)**.

**Figure 6:**
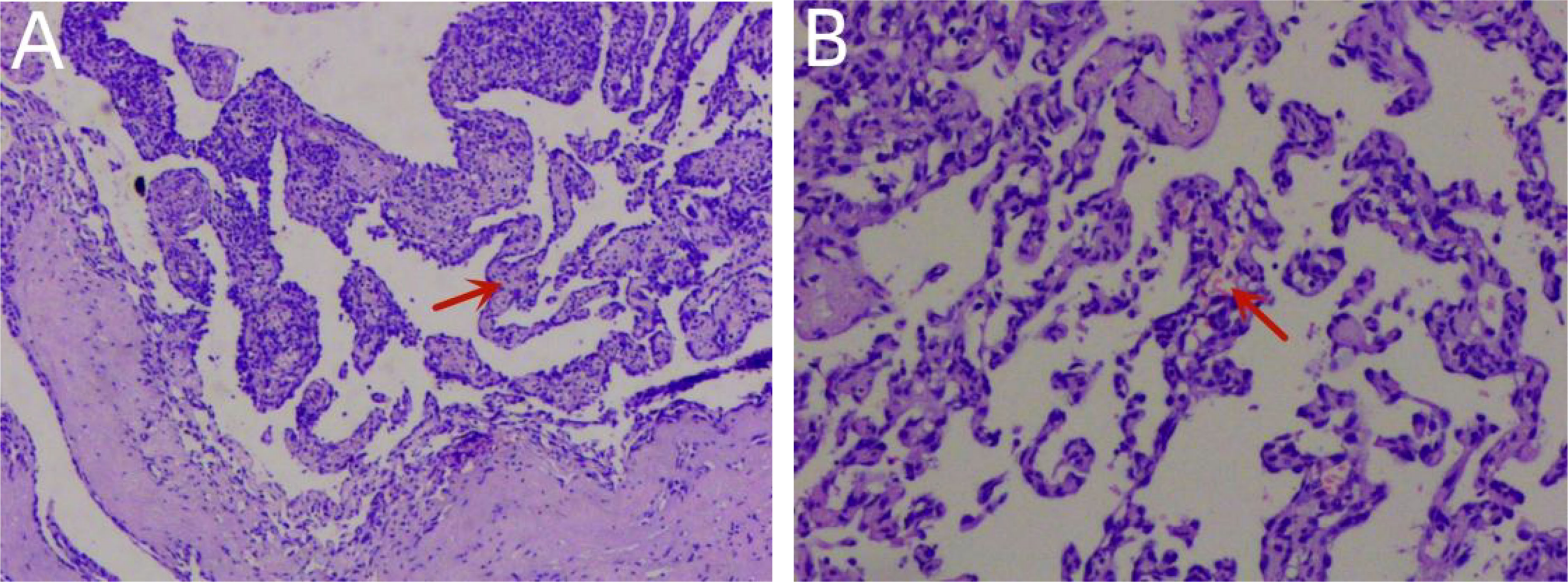
Histopathology confirmed that the tumor was a primary cardiac angiosarcoma **(A)**. Pathological investigation showed vascular hyperplasia, labyrinth and sinusoid structures, obvious cell atypia, mitosis, and massive necrosis in H&E stains **(B)**.

However, the patient continued to experience intermittent shortness of breath after discharge from the hospital, which worsened after exercise. Subsequently, the patient developed symptoms of heart failure due to increased pericardial effusion, and then he was admitted to our hospital for further diagnosis and treatment. After admission, coronary angiography indicated coronary artery sclerosis without obvious stenosis. Therefore, we focused on determining whether the mass was a cardiac tumor or a hematoma and whether it was benign or malignant in the case of a tumor. Subsequently, a right atrial mass resection was performed. A median sternotomy was performed during surgery, followed by a pericardiotomy since the mass was located at the right atrial wall. The serosal surface of the right atrium and superior vena cava showed cowhide changes (**Figure 5A**). A cardiopulmonary bypass was established by blocking the superior and inferior vena cava without obstructing the ascending aorta. Next, the right atrium was dissected, revealing a mass between the walls of the right atrium (**Figure 5B**). The mass of approximately 50 * 40 mm in size started at the junction of the inferior vena cava and atrium and proliferated between the inner and outer membranes of the right atrial wall but did not breach the atrial cavity. According to the tumor morphology, the mass was diagnosed as an atrial sarcoma. The tumor was completely removed, as well as a section of the atrial wall (**Figures 5D, E**), and the right atrial defect was sutured with a pericardial patch (**Figure 5C**). The cardiopulmonary bypass was removed, and the pericardial and mediastinum drainage tubes were positioned. After surgery, the patient was sent to the intensive care unit with endotracheal intubation. Considering the vascular proliferation, labyrinthine and sinus-like angiosarcoma, marked cellular atypia, mitosis, and massive necrosis, the histopathological diagnosis was a tumor of vascular origin (**Figures 6A, B**). Immunohistochemistry showed CD31 (+), CD34 (partial +), ERG (+), FLi-1 (+), and Ki67 (hotspot 30%+). Finally, the patient was discharged after 18 days of hospitalization. At present, 6 months after the surgery, the patient is recovering well.

## Discussion

Primary cardiac angiosarcoma is a rare cardiac tumor (9). Most tumors (approximately 90%) are located in the right atrium, originating from the lateral wall. The left atrium represents the second most frequent location, followed by the right and left ventricles (10, 11). Our patient was a 76-year-old man with a tumor in the right atrium. The clinical symptoms included pericardial effusion and right heart failure, which were consistent with the epidemiological manifestations of angiosarcoma.

Primary cardiac angiosarcoma does not present symptoms during the early stage of the disease, and patients usually manifest overt cardiac symptoms in the later stage (5). The most common symptoms include chest pain, myalgia, palpitations, and dyspnea, which are associated with pericardial effusion, cardiac tamponade, vena cava obstruction, arrhythmias, and chronic heart failure (1, 6). Because angiosarcoma mainly occurs in the right atrium and may invade the pericardium, right ventricle, superior vena cava, and tricuspid valve, it often manifests as right heart failure, superior vena cava obstruction, and pericardial effusion (1, 6, 8, 10). The patient visited the clinic because of chest tightness, fatigue, and dyspnea after activity. The clinical manifestations included bilateral pleural effusion, pericardial effusion, and right heart failure, suggesting that the mass may have invaded the right atrium and pericardium.

The diagnostic approach to cardiac angiosarcoma is mainly based on TTE/TEE, CT, magnetic resonance imaging (MRI), histopathology, and immunohistochemistry (2). In most cases, TTE represents the most convenient preliminary examination since it is accurate, non-invasive, and cost-effective, showing a sensitivity of 75% for primary cardiac angiosarcoma (12). It can describe the size, shape, attachment, and mobility of the tumor, as well as the anatomical relationship between tumors and surrounding structures (7). The TTE of our patient showed an isoechoic, wide-based mass of approximately 39 * 37 mm at the top of the right atrium, multiple echo-free zones, and thickened surrounding atrial walls. However, TEE should also be considered since it presents superior image resolution and better visualization, which can provide more information about the valves, the site of tumor implantation, and wall infiltration, especially those located in the posterior cardiac structure (2, 12, 13). CT and MRI can also be used as adjunctive diagnostic tools for cardiac vascular tumors in addition to echocardiography. These imaging modalities are particularly helpful in defining the extent to which the cardiac tumor infiltrates the surrounding structures and assessing the patient for distant metastases to other organs (5). A plain CT scan of our patient indicated that the angiosarcoma of the heart showed irregular, slightly hypodense shadows with poorly defined boundaries. An improved CT scan showed that most lesions were not enhanced, while the edges appeared locally enhanced. A cardiac MRI can better characterize soft tissue and tumors and exhibits high specificity for pseudotumor, thrombus, and lipoma identification. It is superior in revealing the tumorous infiltration of the myocardium and pericardium but cannot differentiate residual disease (1, 8). In addition, the presence of hemorrhagic pericardial fluid strongly suggested the malignant nature of this tumor. After pericardial puncture and drainage, cytological examination of the pericardial effusion revealed no malignant or atypical cells. Analysis of the pericardial fluid does not always show malignant cells, even in the presence of pericardial invasion, emphasizing the limited diagnostic value of this test (14). Therefore, obtaining a tissue sample is necessary to determine the nature of the heart mass. In this case, the cardiac angiosarcoma diagnosis was confirmed via immunohistochemical staining for endothelial markers, with CD31 a highly sensitive indicator of vascular tumors. ERG oncoprotein, an ETS family transcription factor, is a highly specific and sensitive marker for angiosarcoma (9).

Cardiac angiosarcoma has a poor prognosis due to highly aggressive malignancy, rapid local infiltration and growth, a high rate of systemic metastasis (more than 50% of patients have systemic metastasis at diagnosis) (1, 5), a poor response to adjuvant therapy, a lack of targeted therapy, and other factors (8, 9). Although the most common metastatic site is the lungs, followed by the liver, brain, and bone, metastases to the lymph nodes, pancreas, spleen, mandible, adrenal glands, and kidneys have also been reported (1, 7). According to research by Blackmon SH, the median overall survival (OS) of cardiac sarcoma is 12 months, with 17 months for R0 resection and 6 months for R1 resection. For right heart sarcoma surgery combined with neoadjuvant chemotherapy, the OS is 27 months, and the longest survival time is 9.5 years (2, 15). The survival time of patients will be recorded during follow-up.

No treatment guidelines have been established for primary cardiac angiosarcoma due to its rarity. The current therapeutic approach includes surgery, chemotherapy, and radiation therapy. Surgical resection remains the preferred first-line treatment in the absence of metastasis, and myocardial resection is reparative (1). To improve the chance of survival, surgery requires complete tumor resection. When the tumor cannot be completely resected, partial resection can relieve the hemodynamic obstruction and alleviate short-term clinical symptoms (2, 14). Chemotherapy and radiotherapy are options for patients with incomplete surgical resection. Preoperative chemotherapy may decrease the tumor bulk and eliminate micrometastasis prior to local tumor excision, which is beneficial for patients with metastatic disease (4). For metastatic angiosarcoma, the most eligible drug is doxorubicin combined with ifosfamide. Docetaxel and gemcitabine exhibit similar efficacy for metastatic angiosarcomas. Although high-dose radiation therapy can improve the symptoms caused by local tumors, it can increase serious adverse events (1, 3). Paclitaxel has been used as a radiosensitizer to reduce the adverse effects of high-dose radiation therapy. Combining carboplatin and paclitaxel with radiotherapy has also achieved good therapeutic results (8). Therefore, complete surgical resection combined with chemotherapy and radiation therapy is the preferred treatment for patients with metastasis.

## Conclusion

Primary cardiac angiosarcoma is a rare disease, mostly occurring in the right atrium, which is highly invasive and can cause pericardial and pleural effusion. Due to diagnostic and treatment challenges, most patients have systemic metastases and poor prognoses at the time of diagnosis. The radical resection of the primary tumor remains the most important approach for the optimal survival of patients with early-stage cardiac angiosarcoma without evidence of metastasis.

## Data availability statement

The original contributions presented in the study are included in the article/supplementary material. Further inquiries can be directed to the corresponding author.

## Ethics statement

Written informed consent was obtained from the individual(s) for the publication of any potentially identifiable images or data included in this article.

## Author contributions

HW contributed to the conception of the article. YG acquired the data and wrote the first draft of the manuscript. YG, QL, HW contributed to manuscript revision, read, and approved the submitted version.
